# Antigenic protein modifications in *Ehrlichia*

**DOI:** 10.1111/j.1365-3024.2009.01099.x

**Published:** 2009-06

**Authors:** S THOMAS, N THIRUMALAPURA, E C CROSSLEY, N ISMAIL, D H WALKER

**Affiliations:** Department of Pathology, Center for Biodefense and Emerging Infectious Diseases, University of Texas Medical BranchGalveston, Texas, USA

**Keywords:** *antigen*, Ehrlichia, *ehrlichiosis*, *IOE*, *Eastern blotting*

## Abstract

*To develop effective vaccination strategies against*Ehrlichia*, we have previously reported developing an animal model of cross-protection in which C57BL/6 mice primed with*E. muris *were resistant to lethal infection with*Ixodes ovatus *ehrlichia (IOE). Polyclonal antibody produced in mice after priming with*E. muris *and later injected with IOE-detected antigenic proteins in*E. muris *and IOE cell lysates. Cross-reaction of antigenic proteins was observed when we probed both the*E. muris *and IOE cell lysates with IOE and*E. muris*-specific polyclonal antibody. Analysis of the total proteins of*E. muris *and IOE by two dimensional electrophoresis showed that both*E. muris *and IOE have the same antigenic proteins. Finally, studies on post-translational protein modifications using a novel technique, Eastern blotting, showed that*E. muris *proteins are more lipoylated and glycosylated than those of IOE.*

## INTRODUCTION

The obligate intracellular bacterium *Ehrlichia chaffeensis* that resides in mononuclear phagocytes is the aetiologic agent of human monocytotropic ehrlichiosis (HME). HME is an emerging, highly prevalent, and often life-threatening tick-transmitted infectious disease in the United States (1,2). Development of murine models of persistent ehrlichiosis has greatly facilitated our understanding of the pathogenesis and mechanisms of host defences against primary ehrlichial infections.

Mildly virulent *E. muris* infection in immunocompetent C57BL/6 mice results in persistent infection and mimics *E. chaffeensis* infection in its natural host, white-tailed deer (3), whereas infection of immunocompetent C57BL/6 mice with high dose of a highly virulent *Ehrlichia* species isolated from *Ixodes ovatus* ticks (*I. ovatus* ehrlichia-IOE), mimics severe *E. chaffeensis* infection in humans (4). *Ehrlichia muris* and IOE are antigenically and genetically closely related to each other and to *E. chaffeensis* (5,6). We had previously reported an effective vaccination strategy in a mouse model against fatal ehrlichiosis in which C57BL/6 mice primed with *E. muris* are cross-protected against lethal infection with IOE (4). Furthermore, we showed that prior infection with *E. muris*, but not with IOE, provided protection against lethal secondary IOE challenge. Cross-protection against lethal ehrlichial challenge was associated with substantial generation of IFN-γ-producing memory type 1 CD4^+^ and CD8^+^ T cells, type 1 cytokine production, the development of a strong anamnestic *Ehrlichia*-specific antibody response, and persistent *E. muris* infection. Lack of protection in IOE-primed mice was associated with low frequency of memory type 1 T cells (7).

This study was undertaken to determine which antigens of *E. muris* and IOE are reactive with polyclonal antibody produced in mice after priming with *E. muris* and later superinfected with IOE. Subsequently we observed that *E. muris*- and IOE-specific antibody cross-reacted with IOE and *E. muris* proteins, respectively. Furthermore, we analysed the total proteins of *E. muris* and IOE by two dimensional (2D) gel electrophoresis and found that both *E. muris* and IOE have the same antigenic proteins, but the level of protein modifications was more extensive in *E. muris* than in IOE.

## MATERIALS AND METHODS

### Bacterial culture

Two monocytotropic ehrlichial strains were used in this study, highly virulent *Ehrlichia*spp. (designated IOE) isolated from *I. ovatus* ticks (a gift from Dr M. Kawahara, Nagoya City Public Health Research Institute, Nagoya, Japan) and mildly virulent *E. muris* (provided by Dr Y. Rikihisa, Ohio State University, Columbus, OH). *Ehrlichia muris* was cultivated in DH82 cells at 37°C in DMEM supplemented with 5% heat inactivated bovine calf serum. Ehrlichiae were harvested when approximately 90–100% of the cells were infected. To produce infectious stocks for reproducible studies, C57BL/6 mice were inoculated i.p. with 1 mL of a 10^−1^ dilution (5 × 10^8^ *E. muris*) of the frozen stock. On day 7 after inoculation, the mice were sacrificed, the spleens were harvested, and splenic homogenate was prepared and suspended in DMEM medium. After centrifugation at 11 000 ***g*** the cells were suspended in PBS. The total protein concentrations of the resulting bacterial preparations were determined using a bicinchoninic acid protein assay kit (Pierce, Rockford, IL). DH82 cells or uninfected mouse spleen was used as the negative control.

### Antibodies

For polyclonal antibody production *E. muris* (from infected mouse spleen) was inoculated intraperitoneally into mice and the blood collected on day 45 after the first injection. To generate IOE-specific antibodies we inoculated sublethal doses of IOE at 2 week intervals, and serum was collected after 30 days. For *E. muris/IOE* antibody, mice primed with *E. muris* were infected with IOE on day 30 and the blood collected on day 75 after primary infection.

### Western immunoblots

Total cell lysate from uninfected spleen, spleen infected with *E. muris* and IOE were loaded on to 4–12% Bis–Tris gel (Invitrogen) and the proteins transferred to a nitrocellulose membrane. The membranes were probed with polyclonal sera against *E. muris*, IOE and *E. muris*/IOE (1 : 100 dilution), followed by incubation with goat antimouse alkaline phosphatase (AP). The bands were detected by a chemiluminescence substrate (KPL, MD).

### 2D electrophoresis

For 2D gel electrophoresis, we used an IEF strip (pH 4–7) (NuPage, Invitrogen, CA), followed by separation in a 4–12% Bis-Tris gel. The gels were run according to the protocol of the manufacturers.

### Probes for detecting antigenic protein modification

For detecting lipids, blots were probed with Cholera Toxin B subunit (10 µg/mL) (Sigma, MO), followed by probing with rabbit anti CTB (1 : 5000) and goat antirabbit AP (1 : 1000).

Glucosyl residues were detected using wheat germ agglutinin (WGA) and Con-A. WGA binds Gal NAcα1-ser/thr glycoproteins, whereas Con-A binds glucosyl and mannosyl residues. Biotinylated WGA (0·05 µg/mL) or Con-A (0·005 µg/mL) (Vector Laboratories, CA) was diluted in PBS-BSA3%-Tween 20 and the membrane probed for one hour at room temperature, followed by incubating with Streptavidin-HRP (1 : 15 000). Positive spots were detected by a chemiluminescence substrate (Millipore, MA).

Phosphorylated proteins were detected using the gelcode phosphoprotein detection kit (Pierce-Thermofisher, IL) following manufacturers instruction.

## RESULTS

### Cross-reaction of *Ehrlichia* antibodies

**Figure 1:**
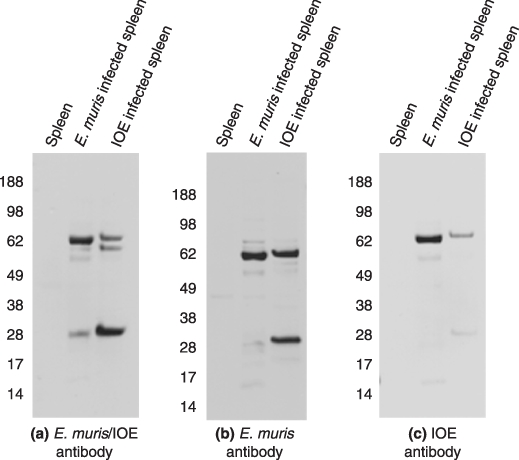
Western blot of one dimensional gel electrophoresis probed with polyclonal antibodies against (a) *E. muris*/IOE (b) *E. muris* and (c) IOE (1 : 100). Five micrograms of cell lysate from mouse spleen, spleen infected with *E. muris* or IOE was used in the study. Representative images based on three independent experiments.

**Figure 2:**
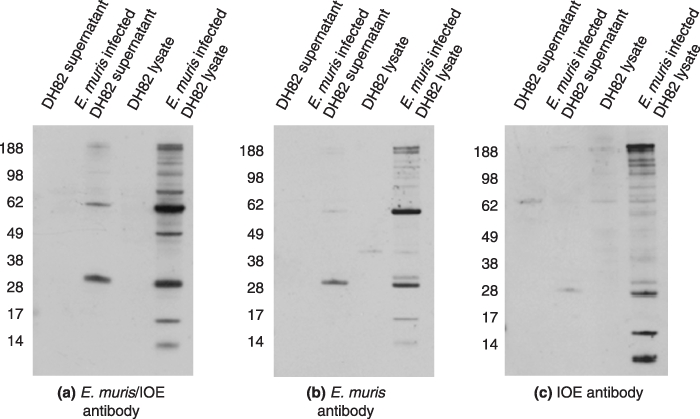
Western blot of one dimensional gel electrophoresis probed with polyclonal antibodies against (a) *E. muris*/IOE (b) *E. muris* and (c) IOE (1 : 100). Five micrograms of cell lysate from supernatant of DH82 cell line, supernatant of DH82 cell line infected with *E. muris*, DH82 cell lysate and *E. muris* infected DH82 cell lysate were used in the study. Representative images based on three independent experiments.

Western blot of one dimensional gel electrophoresis showed that the polyclonal *E. muris/IOE* antibody detected antigenic proteins in both *E. muris* and IOE cell lysates. The predominant antigens were the 60 and 28 kDa proteins. We then explored if the *E. muris* antibody cross-reacted with the IOE proteins. The *E. muris* polyclonal antibody cross-reacted with IOE proteins; similarly, the *E. muris* antigens cross-reacted with the IOE specific antibody (Figure 1). Since the sensitivity of the IOE antibody was less compared to *E. muris* or *E. muris/IOE* polyclonal antibody, we excluded it from further studies. All the three antibodies also detected the antigenic proteins in *E. muris-*infected DH82 cells (Figure 2).

**Figure 3:**
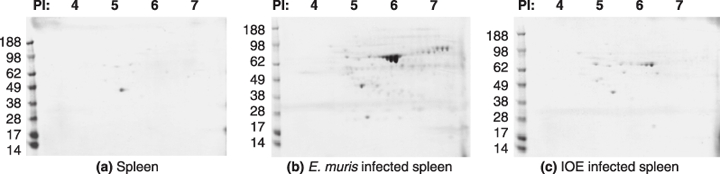
Two dimensional gel of (a) uninfected spleen (b) spleen infected with *E. muris*, and (c) spleen infected with IOE stained with Coomassie blue. Fifty micrograms of protein was used to run the first dimensional gel. Representative images based on three independent experiments.

**Figure 4:**
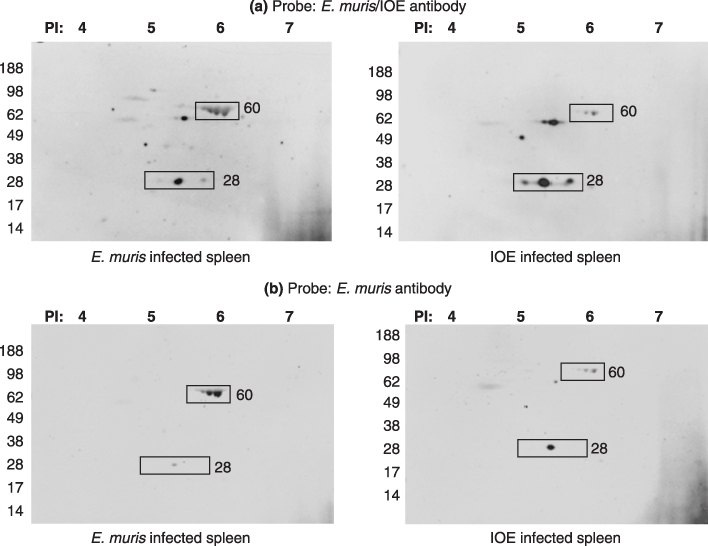
Western blot of two dimensional gels (*E. muris* infected spleen and IOE infected spleen) probed with polyclonal antibodies against (a) *E. muris*/IOE and (b) *E. muris*. Fifty micrograms of protein was used to run the first dimensional gel. The proteins of interest are marked in the image (rectangle).

Coomassie staining of the 2D PAGE gel showed that *E. muris* has more proteins detected than IOE or the uninfected spleen (Figure 3). Both the *E. muris*/IOE and *E. muris* polyclonal antibody detected the *E. muris* and IOE antigenic proteins (Figure 4). The polyclonal antibodies did not detect any antigen in uninfected spleen (data not shown). There was an increase in detection of p28 protein expression in IOE compared to *E. muris* when probed with the *E. muris/*IOE polyclonal antibody; whereas the 60 kDa protein was highly expressed in *E. muris*.

### Detection of protein modifications (Eastern blotting) in antigenic proteins of *Ehrlichia muris* and IOE

It is estimated that 50–90% of proteins are post-translationally modified. These modifications are necessary for the biological functions of a vast array of proteins (8). Studies have suggested important roles for post-translational modifications in a variety of effector-cell functions, including antigen processing, signal transduction and the expression of cytokines and chemokines (9,10). Since the immune recognition of and activation by *E. muris* and IOE are different, we probed for post-translational modifications in the proteins of *E. muris* and IOE.

**Figure 5:**
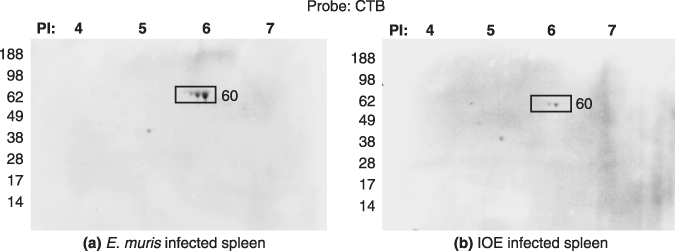
Two dimensional gels of (a) *E. muris* infected spleen and (b) IOE infected spleen probed with Cholera Toxin B subunit for the detection of lipids. Fifty micrograms of protein was used to run the first dimensional gel. Representative images based on three independent experiments. The proteins of interest are shown in boxes.

Cholera toxin is used for the detection of lipids and cholesterol (11,12). We probed the *E. muris* and IOE from infected spleen with cholera toxin B subunit (CTB). The CTB bound to the 60 kDa protein in both *E. muris* and IOE (Figure 5). No spots were observed in the uninfected spleen (data not shown). The 60 kDa protein of *E. muris* was more lipoylated than that of IOE. Lipoproteins are known to induce innate and humoral responses (13), and removal of the lipid moiety from bacterial lipoproteins renders them nonfunctional (14,15).

**Figure 6:**
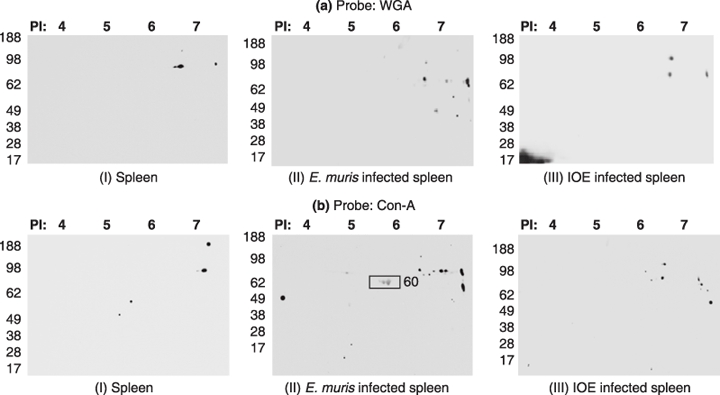
Two dimensional gels of (I) uninfected spleen (II) *E. muris*-infected spleen and (III) IOE infected spleen probed with (a) wheat germ agglutinin and (b) Con-A for the detection of glucose moieties. For the first dimension 50 µg protein was used to run gel. The protein of interest is marked in the image.

WGA a lectin, primarily reacts with N-acetylglucosamine (23 *O*-glycans), whereas Con-A binds to mannosyl and glucosyl residues and both are useful for the detection of glycoproteins. When *E. muris* and IOE were probed with WGA, the probe detected glycoproteins at PI 7·0; whereas, on probing for glycoproteins using Con-A we found that it detected the 60 kDa protein in *E. muris* but not in IOE or the uninfected spleen (Figure 6). Thus in our experiments Con-A was found to be more sensitive for detection of the antigenic glycoprotein. Glycoproteins are known to modulate the immune system and many workers have utilized that property in the production of vaccines (16,17).

**Figure 7:**
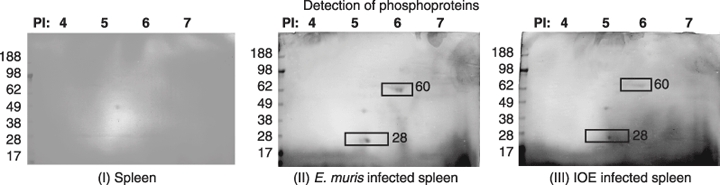
Two dimensional gels of (I) uninfected spleen (II) *E. muris* infected spleen and (III) IOE-infected spleen probed for phosphoproteins. Fifty micrograms of protein was used to run the first dimensional gel. The images are the representative of five independent experiments. The proteins of interest are marked in the images (rectangle).

Knowledge about covalent modifications and their regulation is essential for the understanding of protein function. Regulation of protein activity is often modulated by reversible phosphorylation, and information about specific sites of phosphorylation is vital for understanding cellular signalling pathways. Two-dimensional gel electrophoresis is still the most common method used for detecting large-scale changes in phosphorylation (18). We probed *E. muris* and IOE in 2D gels for phosphorylated proteins and found that both the 60 kDa and 28 kDa proteins of both the species are phosphorylated. Overall, the *E. muris* antigenic proteins were more phosphorylated than those of IOE (Figure 7).

The above results show that the 60 kDa antigenic protein of *E. muris* is more post-translationally modified than the IOE counterpart. Collectively, our data show that the 60 kDa *E. muris* antigenic protein is a phospho-lipo-glycoprotein, whereas in IOE this antigenic protein is a phospho-lipoprotein.

## DISCUSSION

HME manifests as a flu-like illness that can progress in severity to resemble toxic shock-like syndrome, with meningoencephalitis or adult respiratory distress syndrome, and half of patients require hospitalization (1). Currently there are no vaccines against ehrlichial infections. Previous studies in our laboratory in a mouse model have shown that IOE infected mice die within 7–12 days, whereas *E. muris* infected mice are resistant to lethal infection with IOE. In this study we showed that antibodies to both the avirulent *E. muris* and virulent IOE cross-reacted reciprocally with the other ehrlichiae and *E. muris* antigenic proteins had greater post-translation modifications viz., lipoylation, glycosylation and phosphorylation compared to the IOE proteins.

16S rRNA sequences and partial GroEL amino acid sequence of IOE showed the extremely close genetic relatedness of the Anan strain, *E. chaffeensis*, and *E. muris* (19). The 60 kDa protein detected by the *E. muris* and IOE antibody corresponds to GroEL (heat shock protein) (20) and the 28 kDa protein corresponds to the p28 family (21,22). The *groEL* genes, which encode the 60-kDa heat shock protein GroEL, are ubiquitous in both prokaryotes and eukaryotes and encode highly conserved housekeeping proteins which are essential for the survival of these cells (23). In our studies *E. muris* and IOE polyclonal antibodies detected IOE and *E. muris* antigens; hence we presume that both the 60 kDa heat shock protein and p28 are homologous proteins in both the species of *Ehrlichia*.

The technique of gel blotting is useful for visualizing a particular subset of macromolecules – proteins (Western blot), fragments of DNA (Southern blot) or RNA (Northern blot). Since protein modifications are important for signal transduction and it is known that post-translational protein modifications (modified by lipid, glucose or phosphate groups) are immunomodulators and have practical applications for vaccine development we name the technique of detecting protein modifications as Eastern blotting. For our studies we used cholera toxin, Con-A and nitrophospho–molybdate complex for the detection of lipid, glucose and phosphate residues, respectively, on the native proteins of *Ehrlichia*. In this article we showed that the Eastern blotting technique could detect protein modifications in the 60 and 28 kDa proteins of *E. muris* and IOE. The technique shows that among the *Ehrlichia* species *E. muris* proteins have more post-translational modifications than IOE.

The technique of far-Eastern blotting was developed by Ishikawa and Taki (24) as a method for transferring lipids from an HPTLC plate to a PVDF membrane and later probed with antibodies. The technique was also used by Fukuda *et al*. (25) to separate ginsenosides by TLC and blotted to a PVDF membrane treated with NaIO_4_ solution followed by bovine serum albumin (BSA) which resulted in a ginsenoside–BSA conjugate on the PVDF membrane. The blotted spots were finally stained by antiginsenoside monoclonal antibodies. Though the technique used is the same far-eastern technique described by Ishikawa and Taki (24) the authors used the term Eastern blot for their studies. Our technique of Eastern blotting is a simpler technique that proteins blotted from the SDS-PAGE gel to a PVDF or nitrocellulose membrane are analysed for post-translational protein modifications using probes specifically designed to detect lipids, carbohydrate or phospho moieties.

Bacterial lipopolysaccharides (LPS), peptidoglycan, and lipoproteins contribute to pathogenesis by inducing proinflammatory cytokines leading to inflammation and stimulating innate immunity to confer initial host resistance to pathogens (26). The innate immune responses influence the nature of subsequent acquired immune responses, thereby, in combination with inflammation, ultimately affecting host morbidity and mortality. *Ehrlichia* lacks genes for cholesterol biosynthesis (27). A recent study by Huang *et al*. (28) demonstrated that multiple lipoproteins were expressed by *E. chaffeensis* in cell culture. The inhibition of *E. chaffeensis* infection by globomycin (28) or methyl-β-cyclodextrin (27) treatment suggests that lipoproteins are required for *E. chaffeensis* infection of host cells. Lipoproteins are the first components of *E. chaffeensis* shown to induce delayed type hypersensitivity reactions. *Ehrlichia chaffeensis* lipoproteins also induce delayed type hypersensitivity reactions in dogs. Thus, lipoproteins may be considered as potential candidates for a vaccine against HME.

Many bacterial heat shock proteins (GroEL) are immunogenic and are used as vaccine (29,30). GroEL is the predominant antigenic protein in both *E. muris* and IOE and our studies have shown that the protein is phospho-lipo-glycosylated, hence we hypothesize that GroEL is a potential vaccine candidate.

We had reported in previous published articles about the presence of gp140 (5) and gp153 in *E. canis* (31) and gp120 in *E. chaffeensis* (5). We did not detect glycoproteins corresponding to these proteins probably due to species specificity. In addition to the glycosylated 60 kDa we detected glycoproteins of 70 kDa in both *E. muris* and IOE at a PI of 7·0.

Singu *et al*. (20) demonstrated that the p28 outer membrane protein of *E. chaffeensis* is both phosphorylated and glycosylated, whereas in our studies the 28 kDa protein was found to be only phosphorylated in both *E. muris* and IOE.

As recombinant proteins are post-translationally modified by the host bacterium (32) we presume that the technique of Eastern blotting will be able to distinguish protein modifications in recombinant and native proteins. Previous studies have shown that post-translational modifications have the ability to mask epitopes, potentially representing a strategy that pathogens have evolved for their survival in the host (8). As protein post-translational modifications are involved in immune modulation we presume that post-translational modifications observed in both *E. muris* and IOE explain the pathogenecity in mouse model of ehrlichiosis.
